# Editorial Comment: Study of kidney morphologic and structural changes related to different ischemia times and types of clamping of the renal vascular pedicle

**DOI:** 10.1590/S1677-5538.IBJU.2018.0559.1

**Published:** 2019-09-02

**Authors:** Luciano A. Favorito

**Affiliations:** 1 Professor Titular, Unidade Urogenital da Univ. Est. do Rio de Janeiro – UERJ, RJ, Brasil;; 2 Urologista do Hospital da Lagoa Federal, Rio de Janeiro , RJ, Brasil;; 3 Editor Associado do International Braz J Urol Brasil

Mazzeo and collegues from Sao Paulo - Brazil shows in a very interesting paper the morphologic and structural changes of renal parenchyma during the clamping of the renal pedicle. Partial nephrectomy (open, laparoscopic or robotic) is considered the gold standard for treating localized renal tumors (1-6). Warm renal ischemia is commonly performed during partial nephrectomy to achieve a bloodless surgical field, however renal ischemia has been associated with renal function impairment (7).

Previous studies shows that the swine is the most adequate model for comparison to human kidney anatomy and physiology (8, 9). Traditionally, 30 minutes is considered the maximum safe time for renal warm ischemia. In a recent study with swine model (10), the renal warm ischemia of 30 minutes by arterial clamping did not caused significant glomerular damage or nephron loss, but if an artery and vein (*en bloc)* clamping was used, the 30 minutes of warm ischemia caused a decrease in the number of glomeruli.

In the present paper the authors shows that the number of renal parenchymal lesions derived from ischemia is associated with the duration of the insult, but a interesting result was the significant difference between the types of clamping, and the group with clamping of artery and vein presented a lower frequency of injuries than the group with only the renal artery clamping.

According the results of this experimental study during a partial nephrectomy, the en bloc clamping for warm ischemia should be favored over only the renal artery clamping to minimize renal injury after partial nephrectomies, but more studies will be necessary in the future to confirm these results.
